# Impact Of Hypertension versus Diabetes on Cardiovascular and All-cause Mortality in Iranian Older Adults: Results of 14 Years of Follow-up

**DOI:** 10.1038/s41598-017-14631-2

**Published:** 2017-10-27

**Authors:** Neda Zafari, Samaneh Asgari, Mojtaba Lotfaliany, Amirreza Hadaegh, Fereidoun Azizi, Farzad Hadaegh

**Affiliations:** 1 Prevention of Metabolic Disorders Research Center, Research Institute for Endocrine Sciences, Shahid Beheshti University of Medical Sciences, Tehran, Iran; 2 Non-Communicable Disease Control Unit, School of Population and Global Health, University of Melbourne, Victoria, Australia; 3 Endocrine Research Center, Research Institute for Endocrine Sciences, Shahid Beheshti University of Medical Sciences, Tehran, Iran

## Abstract

To evaluate the joint effect of hypertension (HTN) and diabetes (DM) on coronary heart disease (CHD), and stroke event, all-cause, and cardiovascular disease (CVD) mortality in Middle Eastern older adults, 2747 people (1436 women) aged ≥ 50 years, free of CVD at baseline, were categorized into four groups (HTN−/DM−, HTN+/DM−, HTN−/DM+, HTN+/DM+). Multivariate Cox proportional hazard models were run for different outcomes. To compare the impact of HTN versus DM, HTN+/DM− was considered as reference. In a median of 13.9 years, incidence rate of CHD, and stroke event, all-cause and CVD mortality in total population were 19.0, 4.7, 13.5, and 6.4 per 1000 person-years, respectively. The multivariate sex-adjusted hazard ratios (HRs) of HTN−/DM+ for CHD, stroke, all-cause mortality and CVD mortality were 1.19 (confidence interval (CI): 0.9–1.57), 1.07 (CI: 0.63–1.82), 1.62 (CI: 1.2–2.18), and 1.28 (CI: 0.83–1.97); the corresponding HRs for HTN+/DM+ were 1.96 (CI: 1.57–2.46), 1.66 (CI: 1.1–2.52), 2.32 (CI: 1.8–2.98), and 2.6 (CI: 1.85–3.65) respectively. The associations between HTN/DM status with stroke incidence and all-cause mortality were stronger among men than in women (P for interaction <0.05). Compared to HTN+/DM−, HTN−/DM+ increases all-cause mortality by 62%, however, they are not considerably different regarding CHD, stroke incidence and CVD mortality.

## Introduction

The world’s population is aging, (1) a trend that is projected to accelerate in the following decades. The number of elderlies by 2050 is projected to more than double its number in 2015, getting to around 2.1 billion. Likewise, Iran’s elderly population has increased from 7.22% in 2006 to 8.20% in 2011. Having 70.9% of the population in the 15–64 years age group, Iran is awaiting a tremendous rise in its elderly population in the coming decades^1^.

Aging population is at increased risk of developing hypertension (HTN) and type 2 diabetes mellitus (DM)^2–4^, which are two well-known risk factors of cardiovascular disease (CVD)^5^, the leading cause of death worldwide^6^. On one hand, it has been estimated that in 2015, 7.8 million deaths and 143 million disability adjusted life years were related to systolic blood pressures (SBP) ≥ 140 mmHg^7^. On the other hand, DM is considered a coronary heart disease equivalent;^8,9^ and even among Iranian population the population attributed hazard fraction of all-cause mortality for diabetes was greater than the one for prevalent CVD^10^.

Studies have investigated the joint prognostic effect of hypertension and diabetes on CVD risk among American, European and Asian populations^11–14^. Regarding all-cause mortality, data is scarce^14^. Moreover, to the best of our knowledge, there is no evidence regarding the comparative effect of these risk factors on CVD morbidity and mortality in the older adults among the Middle East population which has the highest burden of CVD risk factors and its mortality^15^. To top it off, none of the available studies directly compared hypertensive non-diabetic individuals with diabetic patients who are free of hypertension regarding CVD morbidity and mortality as well as all-cause mortality. Understanding the extent of the association of different combinations of hypertension and diabetes status with CVD events as well as all-cause mortality is crucial especially in low middle income countries where the burden of both the risk factors and outcomes is high, yet, the resources are constrained.

The aim of the current study is to first evaluate the total and sex-specified joint effect of hypertension and diabetes on coronary heart disease (CHD), and stroke event, all-cause, and CVD mortality in older adults in a long-term population based cohort study. Second, to compare the impact of hypertension versus diabetes on the abovementioned outcomes in this population.

## Methods

### Study design and sample

The Tehran Lipid and Glucose Study (TLGS), an ongoing prospective population-based study being performed on a representative sample of the Tehran population, aims to determine the prevalence and incidence of non-communicable diseases and their risk factors. Detailed descriptions of the TLGS have been reported elsewhere^16^. For the current study, 3890 participants, aged ≥ 50 years [3326 people from the baseline examination (1999–2001) and 546 new participants recruited from the second phase (2001–2005)], were selected. Subjects with prevalent CVD at their baseline examination (N = 501), missing data of baseline variables (N = 354) or without any follow-up data after baseline recruitment (N = 288) were excluded; leaving 2747 participants who were followed until 20 March 2014. All experiments were performed in accordance with relevant guidelines and regulations. All subjects filled a written consent after being informed about the general aspects of the work and the study was approved by the Ethical Committee of Research Institute for Endocrine Sciences.

### Clinical and laboratory measurements

Using a pretested questionnaire, a trained interviewer collected information, which included demographic data, smoking and drug history. Physical activity level was assessed with the Lipid Research Clinic (LRC) questionnaire in the first phase of the TLGS. Due to the inexactness of LRC, it was substituted by the Modifiable Activity Questionnaire (MAQ) from the 2nd phase. This questionnaire measures all three forms of activities including leisure time, job, and household activities in the past year^16^.

Weight was measured, with subjects minimally clothed without shoes, using digital scales (Seca 707: range 0–150 kg) and recorded to the nearest 1 kg. Height was measured in a standing position without shoes, using a tape meter, while shoulders were in normal alignment. Waist circumference (WC) was measured at the umbilical level and that of the hip (HC) at the widest girth of the hip over light clothing, using a tape meter, without any pressure to body surface. Measurements were recorded to the nearest 1 cm. BMI was calculated as weight (Kg) divided by height squared (m2). Waist to hip (WHR) was calculated as WC (cm) divided by HC (cm).

After a 15-minute rest in the sitting position, two measurements of systolic and diastolic blood pressure (SBP and DBP) were taken, on the right arm, using a standardized mercury sphygmomanometer (calibrated by the Iranian Institute of Standards and Industrial Researches); the mean of the two measurements was considered as the participant’s blood pressure.

A blood sample was taken between 7:00 and 9:00 AM from all study participants, after 12 to 14 hours overnight fasting. All blood analyses were carried out at the TLGS research laboratory on the day of blood collection. For all non- pharmacologically treated diabetic participants aged 20 years and above, an oral glucose tolerance test with 82.5 g glucose monohydrate solution [equivalent to 75 g anhydrous glucose; Cerestar EP, Spain] was performed and a second blood sample was obtained 2 hours after glucose ingestion. Fasting plasma glucose (FPG) and 2hour-post-challenge plasma glucose (2h-PCPG) were measured using an enzymatic colorimetric method with glucose oxidase; inter- and intra-assay coefficients of variation at baseline and follow-up phases were both less than 2.3%.

Serum total cholesterol (TC) was measured using enzymatic calorimetric tests with cholesterol esterase and cholesterol oxidase. High density lipoprotein cholesterol (HDL-C) was measured after precipitation of the apolipoprotein B containing lipoproteins with phosphotungistic acid. Analyses were performed using Pars Azmon kits (Pars Azmon Inc., Tehran, Iran) and a Selectra 2 auto-analyzer (Vital Scientific, Spankeren, Netherlands). All samples were analyzed when internal quality control met the acceptable criteria.

### Definition of Terms

Education level was classified in 3 categories: i) those who had studied less than 6 years, ii) those who had studied for 6–12 years, and iii) those with more than 12 years of education. Sports or heavy physical activity less than three days per week was defined as low physical activity in the first exam of the TLGS using Lipid Research Clinic (LRC) questionnaire. In the second exam, low physical activity was defined as less than 600 MET (metabolic equivalent of task) per week, using Modifiable Activity Questionnaire (MAQ)^17^. “Daily smoker” was defined as someone who smoked any kind of tobacco at least once a day and “occasional smoker” was one who smoked but not every day; both of them were considered as current smoker. Hypercholesterolemia was defined as total cholesterol ≥ 5.18 mmol/L or taking lipid lowering medications. In accordance with the definition provided by the American Diabetes Association, (29) participants were considered to have T2D if they met at least one of these criteria: FPG ≥ 7.0 mmol/L, or 2h-PCPG ≥ 11.1 mmol/L or taking anti-diabetic medication. According to JNC 7 guideline^18^, hypertension was defined as SBP ≥ 140 or DBP ≥ 90 mmHg or using antihypertensive medication. Participants were categorized into four distinct groups according to their baseline hypertension and diabetes status as followed: i) those who were free of both hypertension and diabetes (HTN−/DM−), ii) non-diabetic hypertensive individuals (HTN+/DM−), iii) diabetic individuals without hypertension (HTN−/DM+), iv) diabetic hypertensive individuals (HTN+/DM+).

### Statistical methods

Mean (SD) values for continuous and frequencies (%) for categorical variables of the baseline characteristics were described. Baseline characteristics were compared between four categories of hypertension and diabetes (HTN−/DM−, HTN+/DM−, HTN−/DM+, HTN+/DM+) using ANOVA or Chi-square tests as appropriate among total population and in each gender.

Multivariate Cox proportional hazard model adjusted for sex (in total population), age, body mass index, waist to hip ratio, education level, low physical activity, current smoking, and hypercholesterolemia was used to evaluate the associations of different combinations of hypertension and diabetes with incident CHD, and stroke events, all-cause and CVD mortality; given HTN−/DM− as the reference. To compare the impact of HTN versus DM, analysis was repeated considering the HTN+/DM− group as reference.

The study survival time was the time from entrance to the study to the date of the first related event. The censoring time of an individual was the time from date of entry into the study until lost to follow-up, death or the study end (20 March 2014), whichever happened earlier.

The proportionality of the multivariable Cox model was evaluated using Schoenfeld’s global test of residuals and all proportionality hypotheses were appropriate. The interactions between gender and different categories of blood pressure and diabetes were checked by log–likelihood ratio test, in multivariate analysis for each event separately. Since there was statistically significant interaction for all-cause mortality (p-value = 0.03) and stroke (p-value = 0.007) analysis was done separately for men and women. However, in order to report comparable results with other studies in this field, we also performed the analysis on a pooled sample.

Statistical analyses were performed using Stata version 12.0 (Stata Corp LP, College Station, Texas).

### Data availability

The datasets generated during and/or analyzed during the current study are available from the corresponding author on reasonable request.

## Results

The study population included a total of 2747 (women = 1436) older adults with mean (SD) age of 59.9 (7.4) years [men: 61.1 (7.8), women: 58.8 (6.8)], and mean (SD) BMI 27.8 (4.5) kg/m^2^. The prevalence of hypertension and diabetes at baseline examination were 43.68% and 23.3%, respectively. Among total population, the prevalence of HTN+/DM−, HTN−/DM+, and HTN+/DM+ were 30.3%, 9.9%, and 13.4%, respectively. All the baseline characteristics except low physical activity in total population were significantly different in four categories. Comparing baseline characteristics between the four categories in men and women revealed a significant difference except for low physical activity in both sexes, education level in men and smoking in women (Table 1).

**Table 1:** Baseline Characteristics* of the Study Population. Tehran Lipids and Glucose Study (1999–2014).

	**HTN (−)/DM (−)**	**HTN (+)/ DM (−)**	**HTN (−)/ DM (+)**	**HTN (+)/ DM (+)**	**P-value**
**Men (N = 1311)**					
*Age*, *year*	59.8(7.7)	63.0(8.0)	61.38(7.8)	62.3(7.3)	<0.001
*BMI*, *kg/m* ^2^	25.4(3.8)	26.9(3.8)	26.9(3.9)	28.3(3.7)	<0.001
*Waist/Hip*	0.94(0.07)	0.97(0.06)	0.97(0.07)	1.0(0.06)	<0.001
*SBP*, *mm Hg*	118.3(11.3)	150.5(17.6)	122.8(11.2)	153.0(22.8)	<0.001
*DBP*, *mm Hg*	74.5(8.3)	90.1(11.1)	75.3(8.1)	90.0(13.3)	<0.001
*Hypercholesterolemia*, *n (%)*	382(56.7)	204(56.4)	94(65.3)	97(74.0)	0.001
*Current smoking*, *n (%)*	185(27.4)	53(14.6)	35(24.3)	21(16.0)	<0.001
*Education*, *n (%)*					0.64
<*6 years*	375(55.6)	218(60.2)	83(57.6)	78(59.5)	
*6–1*2 *years*	217(32.2)	113(31.2)	45(31.2)	41(31.3)	
*≥1*2 *years*	82(12.2)	31(8.6)	16(11.1)	12(9.2)	
*Low physical activity*, *n (%)*	461(68.4)	248(68.5)	97(67.4)	97(74.0)	0.6
*Lipid lowering drugs*, *n (%)*	8(1.2)	15(4.1)	11(7.6)	10(7.6)	<0.001
*Hypertension drug*, *n (%)*	—	88(24.3)	—	37(28.2)	NA
*Diabetes drug*, *n (%)*	—	—	53(36.8)	37(28.2)	NA
**Women (N = 1436)**					
*Age*, *year*	57.3(6.3)	60.0(7.1)	57.5(5.4)	60.8(6.9)	<0.001
*BMI*, *kg/m* ^*2*^	28.25(4.3)	29.7(4.4)	28.6(4.3)	30.5(5.1)	<0.001
*Waist/Hip*	0.88(0.08)	0.9(0.08)	0.92(0.07)	0.94(0.08)	<0.001
*SBP*, *mm Hg*	118.7(11.3)	148.2(20.1)	122.7(10.3)	151.9(20.4)	<0.001
*DBP*, *mm Hg*	75.8(7.6)	90.0(11.1)	77.1(6.8)	90.0(11.6)	<0.001
*Hypercholesterolemia*, *n (%)*	489(81.5)	405(86.0)	107(82.9)	213(90.3)	0.01
*Current smoking*, *n (%)*	29(4.8)	11(2.3)	5(3.9)	5(2.1)	0.09
*Education*, *n (%)*					0.02
<<*6 years*	486(81.0)	403(85.6)	108(83.7)	215(91.1)	
*6–12 years*	97(16.2)	58(12.3)	20(15.5)	19(8.1)	
≥*12 years*	17(2.8)	10(2.1)	1(0.8)	2(0.8)	
*Low physical activity*, *n (%)*	427(71.2)	344(73.0)	91(70.5)	181(76.7)	0.4
*Lipid lowering drugs*, *n (%)*	39(6.5)	46(9.8)	19(14.7)	42(17.8)	<0.001
*Hypertension drug*, *n (%)*	—	207(43.9)	—	126(53.4)	NA
*Diabetes drug*, *n (%)*	—	—	62(48.1)	108(45.8)	NA
**Total population (N** = **2747)**					
*Number (%)*	1274(46.4)	833(30.3)	273(9.9)	367(13.4)	
*Female*, *n (%)*	674(52.9)	362(43.5)	144(52.7)	131(35.7)	<0.001
*Age*, *year*	58.7(7.2)	61.3(7.6)	59.6(7.0)	61.3(7.1)	<0.001
*BMI*, *kg/m* ^*2*^	26.7(4.3)	28.5(4.4)	27.7(4.2)	29.7(4.8)	<0.001
*Waist/Hip*	0.91(0.08)	0.93(0.08)	0.95(0.08)	0.96(0.07)	
*SBP*, *mm Hg*	118.5(11.3)	149.9(19.1)	122.7(10.8)	152.1(21.3)	<0.001
*DBP*, *mm Hg*	75.1(8.0)	90.0(11.1)	76.1(7.6)	90.0(12.2)	<0.001
*Hypercholesterolemia*, *n (%)*	871(68.4)	609(73.1)	201(73.6)	310(84.5)	<0.001
*Current smoking*, *n (%)*	214(16.8)	64(7.7)	40(14.7)	26(7.1)	<0.001
*Education*, *n (%)*					<0.001
<*6 years*	861(67.6)	621(74.5)	191(70.0)	293(79.8)	
*6–12 years*	314(24.6)	171(20.5)	65(23.8)	60(16.3)	
≥*12 years*	99(7.8)	41(4.9)	17(6.2)	14(3.8)	
*Low physical activity*, *n (%)*	888(69.7)	592(71.1)	188(68.9)	278(75.7)	0.13
*Lipid lowering drugs*, *n (%)*	47(3.7)	61(7.3)	30(11.0)	52(14.2)	<0.001
*Hypertension drug*, *n (%)*	—	295(35.4)	—	163(44.4)	NA
*Diabetes drug*, *n (%)*	—	—	115(42.1)	145(39.5)	NA

^*^For continuous variables, mean (SD) is reported; P values for differences across four categories of hypertension and diabetes were calculated using ANOVA test. For categorical variables, frequency is reported; P values for differences across four categories of hypertension and diabetes were calculated using Chi-square test.

HTN, hypertension; DM, type 2 diabetes mellitus; BMI, body mass index; SBP, Systolic blood pressure, DBP, Diastolic blood pressure, NA, not applicable.

In a median (inter-quartile range) of 13.9 (9.8–14.5) years, incidence rate of CHD, and stroke event, all-cause and CVD mortality in total population were 19.0, 4.7, 13.5, and 6.4 per 1000 person years, respectively. Out of 1311 men at baseline, 331 and 90 cases developed CHD, stroke, while 268 and 135 died from all-cause and CVD. Out of 1436 women at baseline, 252 and 64 cases developed CHD and stroke, while 180 and 78 died from all-cause and CVD (Table 2).

**Table 2:** Incidence Rates of Coronary Heart Disease and Stroke and Mortality Rates of Cardiovascular Disease and All-cause in Four Different Categories of Hypertension and Diabetes. Tehran Lipids and Glucose Study (1999–2014).

Event	Coronary Heart Disease Incidence		Stroke Incidence		Cardiovascular Disease Mortality		All-cause Mortality	
	Number of events	Incidence per/1000 person years (95% CI)	Number of events	Incidence per/1000 person years (95% CI)	Number of events	Incidence per/1000 person years (95% CI)	Number of events	Incidence per/1000 person years (95% CI)
**Men**								
**HTN (−)/DM (−)**	132	17.0(14.3–20.2)	18	2.2(1.4–3.4)	31	3.7(2.6–5.3)	77	9.2(7.3–11.5)
**HTN (**+**)/ DM (−)**	102	27.3(22.5–33.1)	37	9.0(6.5–12.4)	45	10.6(7.9–11.3)	89	21.1(17.1–25.9)
**HTN (−)/ DM (**+**)**	43	31.3(23.2–42.2)	13	8.4(4.9–14.4)	22	14.0(9.2–21.3)	48	30.6(23.1–40.6)
**HTN (**+**)/ DM (**+**)**	54	45.8(35.0–59.7)	22	16.5(10.9–25.1)	37	27.0(19.6–37.2)	54	39.5(30.2–51.5)
**Women**								
**HTN (−)/ DM (−)**	58	8.0(6.2–10.3)	18	2.4(1.5–3.8)	10	1.3(0.7–2.5)	44	5.8(4.3–7.9)
**HTN (**+**)/ DM (−)**	83	14.8(11.9–18.4)	25	4.3(2.9–6.3)	30	5.1(3.5–7.2)	59	9.9(7.7–12.8)
**HTN (−)/ DM (**+**)**	28	19.4(13.4–28.1)	5	3.2(1.3–7.7)	7	4.4(2.1–9.3)	16	10.2(6.2–16.6)
**HTN (**+**)/ DM (**+**)**	83	35.04(28.6–43.9)	16	6.0(3.7–9.8)	31	11.4(8.1–16.3)	61	22.5(17.5–28.9)
**Total Population**								
**HTN (−)/ DM (−)**	190	12.6(11.0–14.6)	36	2.3(1.6–3.2)	41	2.6(1.9–3.5)	121	7.6(6.4–9.1)
**HTN (**+**)/ DM (−)**	185	19.8(17.1–22.9)	62	6.2(4.8–8.0)	75	7.4(5.9–9.3)	148	14.6(12.4–17.1)
**HTN (−)/ DM (**+**)**	71	25.21(20.0–31.8)	18	5.8(3.6–9.2)	29	9.2(6.4–13.3)	64	20.4(15.9–26.1)
**HTN (**+**)/ DM (**+**)**	137	38.9(32.9–46.0)	38	9.5(6.9–13.1)	68	16.7(13.1–21.2)	115	28.2(23.5–33.9)

CI, confidence interval; HTN, hypertension; DM, type 2 diabetes mellitus.

**Figure 1:**
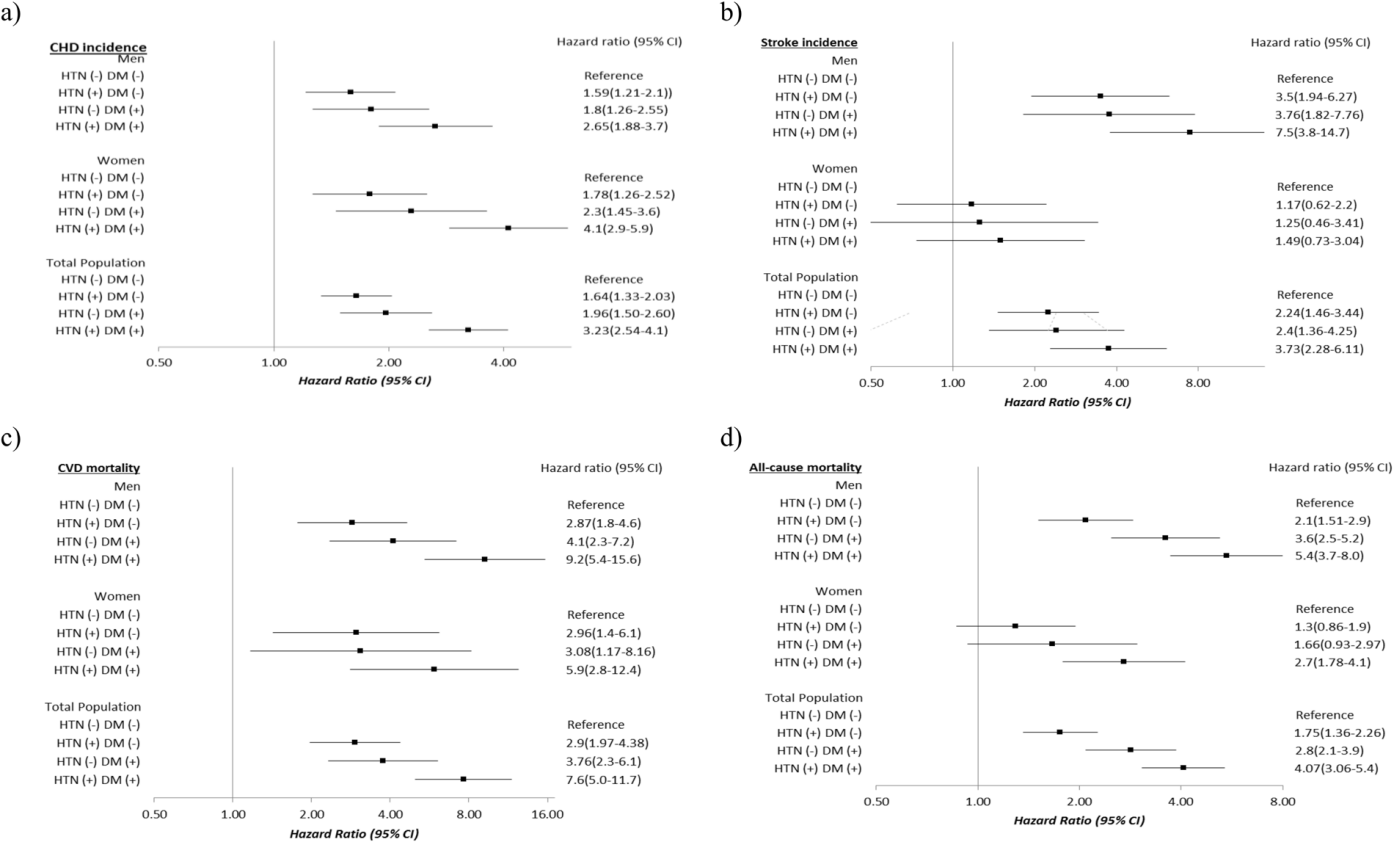
The multivariate adjusted hazard ratio (95% confidence interval) for coronary heart disease and stroke incidence, cardiovascular disease and all-cause mortality. Tehran Lipid and Glucose Study (1999–2014). Multivariate Cox proportional hazard model adjusted for sex, age, body mass index, waist to hip ratio, education level, current smoking, low physical activity, and hypercholesterolemia in 4 different combinations of hypertension and diabetes status i.e. HTN−/DM− (as the reference group), HTN+/DM−, HTN−/DM+, HTN+/DM+, for 4 different outcomes namely (**a**) Coronary heart disease incidence, (**b**) Stroke incidence (**c**) Cardiovascular disease mortality, and (**d**) All-cause mortality. CHD, coronary heart disease; CVD, cardiovascular disease; CI, confidence interval; HTN, hypertension; DM, type 2 diabetes mellitus.

As shown in Fig. 1a looking at the multivariate adjusted hazard ratios for CHD event, in the 3 categories of participants compared to the reference (HTN−/DM−), it was shown that being HTN+/DM− increased the risk for 64% in the total population (59% in men and 78% in women). However, HTN−/DM+ increased the risk for 96% in the total population (1.8-fold in men and 2.3-fold in women). In HTN+/DM+ individuals, the risk of CHD event rose to more than 3-fold [3.23 95% CI (2.54–4.1), 2.65(1.88–3.7), and 4.1(2.9–5.9), in total population, men and women, respectively].

Regarding stroke (Fig. 1b), HTN+/DM− almost doubled the risk of stroke in the total population. The risk increased to more than 3-fold in men [HR (95% CI):3.5, (1.94–6.27)]. HTN−/DM+ showed the HR (95% CI) of 2.4 (1.36–4.25) and 3.76 (1.82–7.76) in the total population and men, respectively. HTN+/DM+ in total population were associated with HR (95%CI) of 3.73 (2.28–6.11). The HR splurged to 7.5 (3.8–14.7) in men. In women, we did not find any risk for different categories of hypertension and diabetes; considering the limited number of events in these groups. In fact, the power of the study to detect the HR of 49% for HTN+/DM+ women was 21%.

Multivariate adjusted hazard ratios (95%CI) for CVD mortality (Fig. 1c) in HTN+/DM− participants were 2.9 (1.97–4.38) in total population, 2.87 (1.8–4.6) in men, and 2.96 (1.4–6.1) in women. HTN−/DM+ had more than 3-fold increased risk in the total population as well as in women, while the number reached to 4-fold in men. In HTN +/DM+ individuals, the risk rose to 7.6-fold in total population, almost 6-fold in women and 9-fold in men.

For all-cause mortality (Fig. 1d) the HR (95%CI) of HTN+/DM− in the total population was 75% (1.36–2.26); the corresponding value for men and women were 2.1 (1.51–2.9) and 1.3 (0.86–1.9). In HTN−/DM+ individuals, the risk increased to 2.8 (2.1–3.9) and 3.6 (2.5–5.2) in total population and men, respectively. HTN+/DM+ had about 4-fold rise (3.06–5.4) among total population; in men and women, the HRs (95% CI) reached to 5.4 (3.7–8.0) and 2.7 (1.78–4.1), respectively.

Repeating the same analyses while choosing HTN+/DM− as the reference group showed that those with HTN−/DM+ were not significantly at greater risk of CHD, stroke and CVD mortality, however, being in the HTN−/DM+ group increased the risk of all-cause mortality for 62% (1.2–2.18) in total population and 72% (1.2–2.45) in men. Those with HTN+/DM+ had increased risk for CHD, stroke, all-cause and CVD mortality compared to HTN+/DM− individuals (Table 3).

**Table 3:** Multivariate Adjusted* Hazard Ratios for Coronary Heart Disease and Stroke Incidence, Cardiovascular Disease and All-cause Mortality According to Hypertension and Diabetes Status. Tehran Lipids and Glucose Study (1999–2014).

	CHD Incidence	P-value	Stroke Incidence	P-value	CVD Mortality	P-value	All-cause Mortality	P-value
**Men**								
**HTN**+**/DM−**	Reference	—	Reference	—	Reference	—	Reference	—
**HTN−/DM**+	1.13(0.79–1.62)	0.5	1.08(0.57–2.04)	0.82	1.43(0.85–2.39)	0.18	1.72(1.2–2.45)	0.003
**HTN+/DM+**	1.67(1.19–2.34)	0.003	2.14(1.23–3.72)	0.007	3.21(2.03–5.06)	<0.001	2.61(1.83–3.71)	<0.001
**HTN−/DM−**	0.63(0.48–0.82)	0.001	0.29(0.16–0.52)	<0.001	0.34(0.21–0.56)	<0.001	0.48(0.35–0.66)	<0.001
**Women**								
**HTN**+**/DM−**	Reference	—	Reference	—	Reference	—	Reference	—
**HTN−/DM**+	1.28(0.83–1.97)	0.26	1.07(0.4–2.86)	0.89	1.04(0.45–2.41)	0.92	1.28(0.73–2.26)	0.38
**HTN+/DM+**	2.31(1.69–3.15)	<0.001	1.28(0.67–2.42)	0.45	1.99(1.20–3.31)	0.008	2.09(1.45–3.01)	<0.001
**HTN−/DM−**	0.56(0.4–0.79)	0.001	0.85(0.45–1.61)	0.63	0.34(0.16–0.70)	0.004	0.77(0.51–1.16)	0.21
**Total Population**								
**HTN+/DM−**	Reference	—	Reference	—	Reference	—	Reference	—
**HTN−/DM**+	1.19(0.9–1.57)	0.21	1.07(0.63–1.82)	0.8	1.28(0.83–1.97)	0.27	1.62(1.2–2.18)	0.001
**HTN+/DM+**	1.96(1.57–2.46)	<0.001	1.66(1.1–2.52)	0.017	2.6(1.85–3.65)	<0.001	2.32(1.8–2.98)	<0.001
**HTN−/DM−**	0.61(0.49–0.75)	<0.001	0.44(0.29–0.68)	<0.001	0.34(0.23–0.51)	<0.001	0.57(0.44–0.73)	<0.001

*Adjusted for sex (for total population), age, BMI, waist to hip ratio, education level, current smoking, low physical activity, and hypercholesterolemia.

CHD, coronary heart disease; CVD, cardiovascular disease; HTN, hypertension; DM, type 2 diabetes mellitus.

Standard errors were reported as confidence intervals and P-values.

## Discussion

Conducted among the Iranian adults aged 50 years or above during a more than a decade follow-up, the current study showed that compared to those who were HTN−/DM− at baseline, both hypertension and diabetes either alone or accompanied together increased the risk of CHD event, all-cause and CVD mortality both in men and women. Stroke incidence was positively associated with hypertension and diabetes (alone or together) in men as well as sex-adjusted multivariate models in the total population. Having HTN+/DM− individuals as the reference group, the all-cause mortality in HTN−/DM+ group was increased for 72% in men and 62% in total population.

The independent impact of hypertension and diabetes on CHD, stroke, all-cause and CVD mortality in older adults has been shown in many studies^4,14,19–25^. As shown in many studies using HTN−/DM− as reference, we illustrated that HTN+/DM+ individuals had the highest risk of developing CHD, and stroke as well as death from all-cause and CVD^26–28^. A large cohort study conducted among Finnish population^11,12^, showed that those with both hypertension and diabetes at baseline examination were at highest risk for incident CHD, stroke and their mortalities; furthermore, Kokubu *et al*. showed the same results regarding CVD events. However, these findings were not obtained among the American population^13^. We extend the previous studies by showing individuals with both risk factors had higher risk for different outcomes compared with hypertensive non-diabetic individuals in multivariate sex adjusted analysis. This is important especially due to the fact that incidence of hypertension in diabetic patients and diabetes in hypertensive individuals increases with aging^26,29^.

Importantly, among hypertensive individuals in our study, only 38% consumed antihypertensive drugs at baseline examination. Results of a cohort study conducted in northern region of Iran reported that merely17% of hypertensive subjects were using antihypertensive medications;^30^ a number that reached 25% in national surveys^31^. Regarding diabetic individuals, in the current study, 40% consumed anti-diabetic medications. Similarly, previous studies in TLGS population showed that only half of diagnosed diabetic patients used anti-diabetic treatment^32^. Moreover, national surveys estimate that about half of the diabetes cases are undiagnosed^33^. Hence, it might be hypothesized that, the low frequency of antihypertensive and anti-diabetic drugs consumption in our population might be attributable to low awareness of the disorders in the population, inertia for initiating treatment and low adherence to treatment regimen^34–36^. This issue may contribute to the higher risk of CVD morbidity and mortality among hypertensive and diabetic individuals^37^.

To the best of our knowledge, our study is the first to conduct joint effect analysis comparing HTN−/DM+ individuals with HTN+/DM− persons. There are few studies conducted among European, American and Asian examining different impact of hypertension and diabetes on CVD incidence and mortality^11–14^, however, all of them considered HTN−/DM− participants as the reference group. Moreover, only one study assessed this issue regarding all-cause mortality^14^. We found out that there is no statistically significant difference between HTN−/DM+ and HTN+/DM− individuals in developing CHD, stroke and CVD mortality, however, the former group showed more than 60% higher risk for all-cause death compared to the latter in the total population as well as men. In a recent study, Oh *et al*. argued that in American older adults, compared to diabetes, hypertension has a stronger association with all-cause and CVD mortality. Nevertheless, their conclusion might not be appropriate due to the overlapping confidence intervals of HTN−/DM+ and HTN+/DM− groups^14^. In line with our results, in the study published by Hu *et al*. the HR for CHD incidence in Finnish diabetic men and women who were free of hypertension was 2.39 (1.61–3.55) and 5.63 (3.20–9.88), respectively; the corresponding values for non-diabetic stage1 hypertensive individuals were 1.25 (1.13–1.39) in men and 1.52 (1.28–1.81) in women; all of which had CIs far enough from the former group. Even the risk for diabetes was higher than among non-diabetic stage 2 hypertensive women^12^. Regarding stroke, results were generally the same for stage 1 hypertension^11^. Several reasons could explain our finding; first, diseases such as cancer and autoimmune disorders may induce insulin resistance^38^. Thus, those with such pre-existing conditions may increase the all-cause mortality under the name of diabetes. For this issue, when we excluded the event mortality during the first 3 years of follow-up, results did not change (data not shown). Hence, the reverse causality was not responsible for the current results. Second, diabetes complications may have been considered independent causes of death. A recent meta-analysis proved that apart from increasing the risk of vascular diseases, diabetes is associated with substantial premature mortality from several cancers, infectious diseases, external causes, and intentional self-harm, and degenerative disorders, independent of several major risk factors including systolic blood pressure^39^.

Considering limitations, first, as inherent to any prospective study, the level of risk factors at the baseline examination might change during the follow up. Second, the power of the study to detect the HR for stroke incidence in women was low. Third, unfortunately, measurements of HbA1C were not performed for participant of TLGS; hence, we could not use HbA1c as a criterion for defining diabetic patients. Finally, the present study was carried out among Persian ethnicities, so the present results cannot be directly extrapolated to other populations.

As for the strengths, our study is the first studying the joint effect of hypertension and diabetes in a large representative sample of Iranian older adults in a population with reliable follow-up data. We had reasonable number of events in the total population which allowed us to evaluate the long term effects of hypertension and diabetes on CHD, stroke incidence and all-cause and CVD mortality. Also, we used both FPG and 2h-PCPG as indicators of diabetes status allowing us to have an accurate estimation of incident T2D.Last but not least, considering HTN+/DM− participants as the reference group; we evaluated the impact of diabetes versus hypertension on a variety of outcomes.

To the best of our knowledge, our study is the first investigating the joint effect of two worldwide common risk factors of CVD i.e. hypertension and diabetes considering HTN+/DM− people as the reference group, hence, comparing HTN−/DM+ individuals directly with the former group. We showed that the all-cause mortality in HTN−/DM+ people in the total population increased for 62%; a number that rose to 72% in men. Further studies are needed to figure out the impact of hypertension versus diabetes on CVD events as well as all-cause mortality in other ethnicities.
